# Three-Dimensional Perspectives on Inflammatory Regulation in Coronary Atherosclerosis: Integrated Mechanisms of Endothelial Priming, Lipid Metabolism, and Cytokine Synergy

**DOI:** 10.31083/RCM42822

**Published:** 2026-01-19

**Authors:** Min Liu, De-Gang Mo, Jing-Xian Bai, Qian-Feng Han, Heng-Chen Yao

**Affiliations:** ^1^Shandong First Medical University, 250117 Jinan, Shandong, China; ^2^Department of Cardiology, Liaocheng People's Hospital, 252000 Liaocheng, Shandong, China; ^3^School of Medicine, Qingdao University, 266000 Qingdao, Shandong, China

**Keywords:** atherosclerosis, endothelial dysfunction, dysregulated lipid metabolism, inflammation, biomarker

## Abstract

Atherosclerosis, a leading cause of global mortality, is a chronic inflammatory disease driven by a vicious cycle of endothelial dysfunction, dysregulated lipid metabolism, and persistent inflammation. This review examines the mechanisms through which diverse triggers initiate the cycle. We discuss key cellular and molecular events, such as the detrimental phenotypic switching of vascular smooth muscle cells. We also describe the processes through which various upstream signals converge on core inflammatory hubs, such as the Toll-like receptor 4 (TLR4)/nuclear factor-κB (NF-κB) pathway and the nucleotide-binding oligomerization domain, leucine-rich repeat-containing family, pyrin domain-containing-3 (NLRP3) inflammasome. By integrating these established mechanisms with recent findings on novel regulators, including the chemokine hemofiltrate CC chemokine 1 (HCC-1) and cell surface glycoRNA, this review identifies several potential new biomarkers. Overall, this review aimed to provide a comprehensive understanding of the pathogenesis of atherosclerosis, informing future research and the development of targeted interventions.

## 1. Introduction

Cardiovascular diseases (CVDs) are the leading cause of global mortality, with coronary artery disease (CAD) being the most common form. CAD is caused by atherosclerosis and was responsible for 9.44 million deaths and 185 million disability-adjusted life years (DALYs) in 2021, according to the Global Burden of Disease study [1]. DALYs is a composite measure of disease burden that combines years of life lost due to premature mortality (YLL) and years lived with disability (YLD).

Atherosclerosis is fundamentally a chronic inflammatory disease characterized by lipid deposition and plaque formation in the artery wall [2]. Its progression is driven by a vicious cycle involving three core processes. The cycle begins with endothelial dysfunction, which allows lipids such as low-density lipoprotein (LDL) to be retained in the arterial wall. This lipid accumulation then triggers a persistent inflammatory response that, in turn, worsens endothelial function and lipid handling, driving plaque growth and eventual rupture [3].

Understanding the synergy between endothelial dysfunction, lipid metabolism, and inflammation is critical for the development of more effective therapies. This review systematically explores the interplay of these three core mechanisms in the inflammatory regulation of atherosclerosis, integrating emerging research with established pathways. The goal is to provide new perspectives on coronary atherosclerosis and inform the development of novel biomarkers and targeted treatments.

## 2. Endothelial Dysfunction and Inflammation

**Fig. 1.**
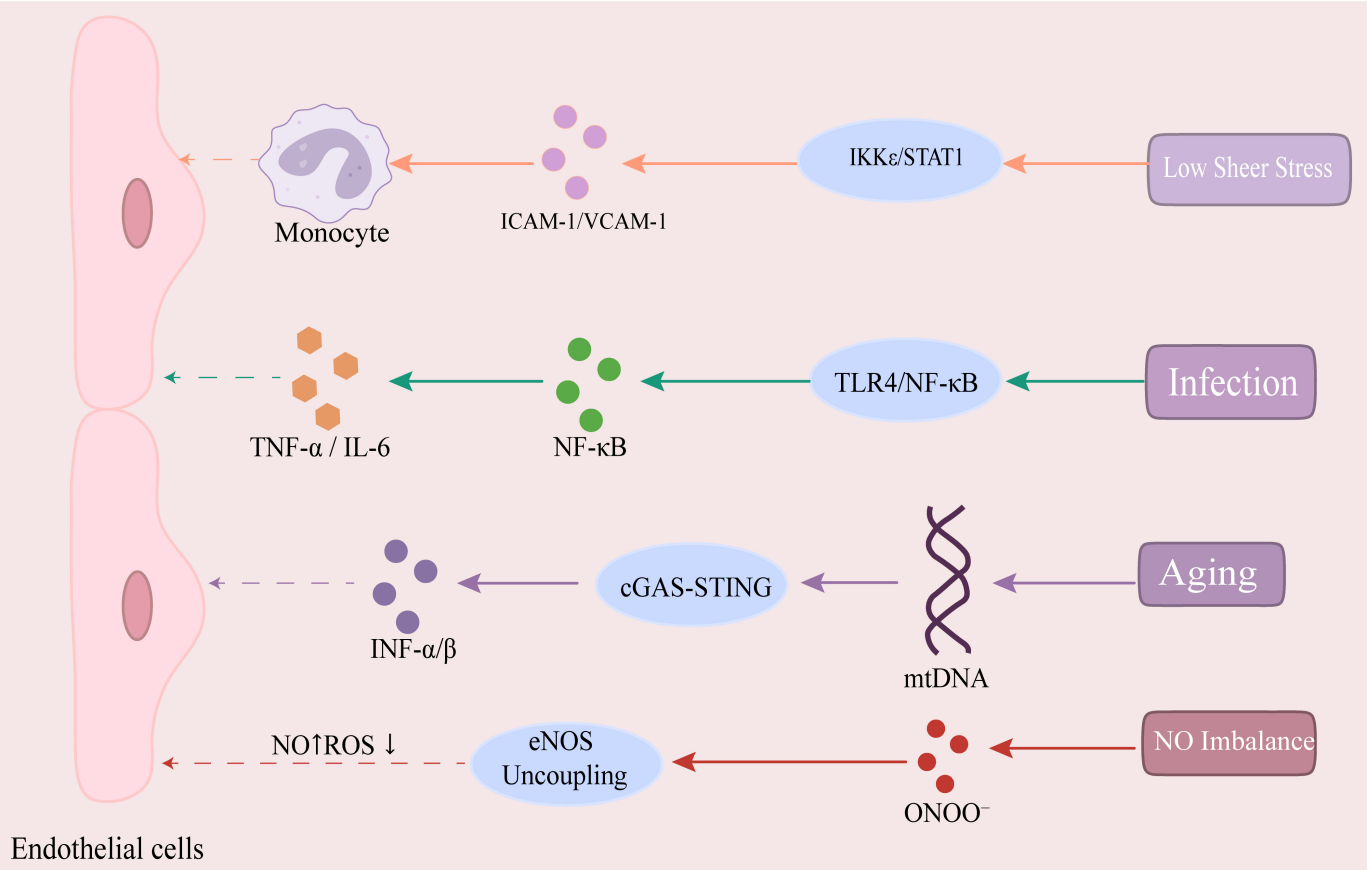
**Key pathways in endothelial cell dysfunction**. Low shear stress (LSS) activates the IκB kinase epsilon (IKKε)/signal transducer and activator of transcription 1 (STAT1) pathway, upregulating the expression of intercellular adhesion molecule 1 (ICAM-1), vascular cell adhesion molecule 1 (VCAM-1), and monocyte chemoattractant protein-1 (MCP-1, also known as CCL2), thereby promoting monocyte infiltration and exacerbating endothelial dysfunction. Infectious agents activate the extracellular signal-regulated kinases 1 and 2 (ERK1/2)/STAT1 signaling axis by binding to Toll-like receptor 4 (TLR4) and recruiting the adaptor proteins myeloid differentiation primary response 88 (MyD88, an adaptor protein) and TIR-domain-containing adapter-inducing interferon-β (TRIF, an adaptor protein) to initiate downstream signaling. MyD88-dependent activation of the TNF receptor-associated factor 6 (TRAF6) and IKK complex phosphorylates IκBα, releasing nuclear factor-κB (NF-κB) for nuclear translocation, where it drives the expression of pro-inflammatory factors such as TNF-α, IL-6, IL-1β, and chemokines (chemokine C-C motif chemokine ligand 2 (CCL2), C-X-C motif chemokine ligand 8 (CXCL8, widely known as IL-8)), thereby exacerbating endothelial injury. Under conditions of oxidative stress, excess reactive oxygen species (ROS) can directly scavenge NO molecules to form peroxynitrite (ONOO⁻), which induces conformational changes in endothelial nitric oxide synthase (eNOS) that lead to its uncoupling. This leads to impaired vasodilation and aggravated inflammation. Aging-related mitochondrial dysfunction results in the leakage of mitochondrial DNA (mtDNA), triggering activation of the cyclic GMP-AMP synthase (cGAS)-stimulator of interferon genes (STING) pathway which then induces type I interferon (interferon–α/β)-driven sterile inflammation and suppresses peroxisome proliferator-activated receptor-gamma coactivator (PGC)-1α-mediated mitochondrial biogenesis. This results in a sustained reduction in eNOS activity and promotes the release of senescence-associated secretory phenotype (SASP) factors (IL-6, TGF-β), fostering a pro-inflammatory microenvironment. Fig. 1 was generated utilizing Adobe Illustrator 2025.

Endothelial cells (ECs) form a continuous monolayer lining the vascular lumen, serving as a biological barrier that precisely regulates the transmembrane transport of nutrients and signaling mediators. Endothelial dysfunction is a core pathological event in atherosclerosis [4] and is characterized by reduced biosynthesis of endothelium-derived nitric oxide (NO) and accumulation of reactive oxygen species (ROS). This leads to impaired vasodilation, activation of a pro-inflammatory phenotype, and an imbalance between procoagulant and anticoagulant activities, significantly increasing the risk of major adverse cardiovascular events (MACE). EC dysfunction can be triggered by various factors that compromise the endothelial barrier through specific signaling pathways, leading to monocyte infiltration [5]. The relevant factors and mechanisms are shown in Fig. 1.

### 2.1 Mechanisms That Trigger Endothelial Dysfunction

Endothelial activation is a key hallmark of dysfunction, primarily driven by pro-inflammatory cytokines such as TNF-α and IL-6. This state is characterized by the overexpression of adhesion molecules and procoagulant factors (e.g., vascular cell adhesion molecule 1 (VCAM-1), intercellular adhesion molecule 1 (ICAM-1), E-selectin) [6], which promote the adhesion of circulating immune cells to the vascular wall, thereby initiating early atherosclerotic lesions.

#### 2.1.1 Shear Stress

Low shear stress (LSS) is recognized as a critical initiator of endothelial dysfunction. LSS is a pathological state in which the frictional force of blood flow on the vessel wall is significantly reduced. This condition occurs most commonly at vascular bends and bifurcations and is an important indicator of hemodynamic disturbance [7]. LSS directly alters endothelial cell structure and function and stimulates the migration and proliferation of vascular smooth muscle cells and monocytes [8]. LSS activates the IκB kinase epsilon (IKKε)/signal transducer and activator of transcription 1 (STAT1) pathway, with the specific mechanisms shown in Fig. 1. It also triggers assembly of the nucleotide-binding oligomerization domain, leucine-rich repeat-containing family, pyrin domain-containing-3 (NLRP3) inflammasome to induce the release of pyroptosis-related proteins (gasdermin D, IL-1β). Furthermore, LSS increases oxidative stress by elevating ROS levels and suppressing antioxidant genes, all of which reduce the bioavailability of protective NO [9, 10, 11, 12].

Emerging evidence shows that shear stress can affect endothelial autophagy, with defective autophagy subsequently promoting atherosclerosis. Studies using cellular and animal models have suggested the mechanosensitive channel Piezo1 is activated under LSS and oxidized low-density lipoprotein (ox-LDL) stimulation. This leads to the nuclear translocation of Yes-associated protein (YAP), which in turn inhibits autophagy [13]. Another preclinical investigation has highlighted the potential role of SRY-related High Mobility Group box transcription factor 4 (SOX4), which was observed to be highly expressed in vascular areas with LSS in both human tissues and in mouse models. Supporting this observation, the experimental overexpression of SOX4 in isolated mouse ECs and aortic roots resulted in the loss of endothelial markers. In a separate *in vitro* model, treatment with the antidiabetic drug metformin was shown to reverse cytokine-induced SOX4 expression in human umbilical vein ECs (HUVECs). Collectively, these preclinical data suggest that SOX4 is a potential regulator, but its definitive role in human disease requires further validation [14].

Beyond its effects on autophagy, disturbed blood flow also promotes atherosclerosis by inducing the protein CCN1 (also known as cysteine-rich angiogenic inducer 61). *In vitro* studies have demonstrated that oscillatory shear stress significantly upregulates CCN1 expression in HUVECs and mouse aortic ECs. This upregulation helps create a positive feedback loop whereby CCN1 and integrin α6β1 activate nuclear factor-κB (NF-κB), which in turn amplifies their own expression and perpetuates endothelial dysfunction [15]. The entire process is reinforced by the mechanosensor YAP, which is also activated by disturbed flow to drive CCN1 expression and an atherosclerotic phenotype [16]. In contrast, steady, laminar shear stress is generally protective against atherosclerosis.

#### 2.1.2 Infection

Beyond physical stresses like disturbed blood flow, infectious agents are another major cause of endothelial damage. General viral infections can activate macrophages to release inflammatory cytokines (e.g., TNF, IL-6), which disrupt endothelial tight junctions and degrade the vascular basement membrane by upregulating trypsin and matrix metalloproteinase-9 (MMP-9), leading to increased vascular permeability and inflammation [17]. Following Influenza A virus (IAV) infection, elevated levels of IAV mRNA and viral antigens were observed in the arterial wall and perivascular adipose tissue (PVAT) of pregnant mice. IAV induced expression of the antiviral mediator IFN-γ, which was associated with vascular endothelial dysfunction [18]. The dengue virus replicates within ECs, inducing cell death (apoptosis) via a caspase-3-dependent pathway and releasing high mobility group box 1 (HMGB1), which disrupts the integrity of the endothelial barrier [19]. Similarly, the SARS-CoV-2 spike protein has been shown to cause endothelial damage through multiple mechanisms. In studies [20] on infected mouse brain microvascular ECs, the spike protein induced degradation of key junctional proteins, impairing barrier function. Furthermore, *in vitro* experiments using human aortic ECs (HAECs) demonstrated that treatment with recombinant spike protein increased the secretion of inflammatory and pro-thrombotic markers [20].

*In vitro* cellular experiments have also revealed that Gram-negative bacterial endotoxin (LPS) induces ROS accumulation, which activates the ERK1/2/STAT1 pathway and upregulates inflammatory molecules such as VCAM-1 and various cytokines. At the same time, LPS increases the expression of HMGB1 and the receptor for advanced glycation end-products (RAGE). The subsequent secretion of HMGB1 and its binding to RAGE disrupts EC-cell junctions, thereby promoting atherosclerosis [21]. During the immune response against pathogens, the phenomenon of molecular mimicry may induce cross-reactivity, causing the immune system to mistakenly target structurally similar self-proteins within the vascular wall, thereby triggering chronic autoimmune-mediated vascular injury [22].

These preclinical findings, which link pathogens to vascular damage, are echoed in clinical observations. Data from a multicenter registry indicate that patients with ST-segment elevation myocardial infarction (STEMI) during the first wave of the COVID-19 pandemic experienced longer ischemic times and higher rates of adverse events [23]. However, this clinical data does not establish a direct causal relationship between the worse outcomes and accelerated atherosclerosis in these patients.

#### 2.1.3 NO Metabolism

By disrupting NO metabolic homeostasis, oxidative stress becomes a core driver of endothelial dysfunction. Under physiological conditions, ECs catalyze the conversion of L-arginine to L-citrulline via endothelial nitric oxide synthase (eNOS), synthesizing NO molecules with vasodilatory and anti-inflammatory properties. The activity of eNOS is dually regulated by calcium-dependent phosphorylation (e.g., Akt-mediated modification at Ser1177) and calcium-independent mechanisms (e.g., binding to heat shock protein 90) [24]. However, under conditions of oxidative stress, eNOS becomes uncoupled, reducing NO production and increasing ROS [25]. This “NO-ROS imbalance” impairs the vasodilation capacity and promotes the expression of inflammatory factors via the NF-κB pathway, creating a vicious cycle of endothelial damage.

Additionally, oxidative stress inhibits the bioavailability of tetrahydrobiopterin (BH4). BH4 is an essential cofactor for electron transfer in the eNOS catalytic cycle. Under oxidative stress, excess O^2-^ oxidizes BH4 to BH2, which can competitively replace BH4 and weaken its role in eNOS catalysis. This ultimately results in reduced NO synthesis and dysfunction of eNOS. However, in patients with CAD, direct supplementation with BH4 analogs failed to improve endothelial function and was associated with an increase in BH2 levels [26].

Recent studies have identified vascular endothelial protein tyrosine phosphatase (VE-PTP) as a key regulator of endothelial homeostasis. This enzyme negatively modulates eNOS activity by dephosphorylating members of signaling complexes such as the Tie-2 receptor, CD31, VE-cadherin, and vascular endothelial growth factor receptor 2 (VEGFR2, a key receptor tyrosine kinase) [27]. Oxidative stress can upregulate VE-PTP expression. The resulting dephosphorylation inhibits the Tie-2/Akt/eNOS signaling axis, which reduces NO synthesis and causes abnormal vascular tone and endothelial barrier disruption. Targeted inhibition of VE-PTP has been shown to restore eNOS function, improve endothelium-dependent vasodilation, and reduce vascular resistance in animal models of hypertension [28]. These observations suggest that VE-PTP is a potential therapeutic target, although it remains to be determined whether such benefits are directly transferable to the distinct pathological context of atherosclerosis.

#### 2.1.4 Aging

The intrinsic process of aging is a primary driver of endothelial dysfunction, as demonstrated in aged mice and *in vitro* models using HAECs. With advancing age, a study has shown that protective eNOS expression decreases, whereas aging markers (e.g., p53, p21) and components of the cyclic GMP-AMP synthase (cGAS)-stimulator of interferon genes (STING) pathway increase. Notably, inhibiting the cGAS-STING pathway reversed these changes in both animal and cellular models. Mechanistically, this process is driven by the cGAS-STING pathway, which acts as a sensor for age-related damage. During senescence, DNA leaks within the cell are sensed by cGAS, which in turn activates STING. Activated STING subsequently induces type I interferon production, leading to a state of sterile inflammation that further accelerates endothelial senescence [29].

In addition to the cGAS-STING pathway, abnormal activation of RhoA/Rho kinase (ROCK, a key downstream effector of the small GTP-binding protein RhoA) signaling is another central mechanism in aging-related endothelial damage. ROCK activation in the vasculature can disrupt the VE-cadherin/β-catenin complex by phosphorylating myosin light chain (MLC) to increase vascular permeability. ROCK activation also stimulates nicotinamide adenine dinucleotide phosphate oxidase (NOX) 2/4 to promote oxidative stress via ROS production, as well as inducing EC apoptosis [30]. Under aging stress conditions, RhoA can compete with eNOS for the substrate L-arginine by activating arginase I and synergizing with LPS-Toll-like receptor 4 (TLR4) signaling to amplify NF-κB-mediated inflammatory responses [31]. However, the role of RhoA/ROCK is complex and context-dependent, particularly in the heart. Paradoxically, one study has shown that a deficiency of RhoA in the heart can cause premature cardiac aging and heart failure, as the pathway is essential for maintaining mitochondrial health through mitophagy [32]. Further complicating this, deletion of only the ROCK2 isoform in cardiomyocytes was also found to be detrimental by promoting fibrosis and reducing autophagy. These findings challenge the simplistic view that ROCK activation is uniformly harmful in all cardiovascular tissues.

Further research into the mechanisms of vascular aging has identified the RNA-binding protein Grb10-interacting GYF protein 2 (GIGYF2) as a key regulator. GIGYF2 is overexpressed in senescent human ECs and in the aortas of aged mice. Overexpression of GIGYF2 in young ECs induces senescence, while silencing or knocking out GIGYF2 in aged cells and mice reduces senescence markers and improves vascular function by enhancing NO production. Mechanistically, GIGYF2 stabilizes Staufen double-stranded RNA binding protein 1 (STAU1) mRNA, increasing its protein translation and thereby activating the mechanistic target of rapamycin complex 1 (mTORC1)/ribosomal protein S6 kinase 1 (S6K1) signaling axis. Activated mTORC1 then inhibits autophagy, causing abnormal protein aggregation, and impairs Sirtuin1 (SIRT1)-dependent eNOS function [33]. This cascade accelerates endothelial aging and dysfunction, suggesting that targeting the GIGYF2-STAU1-mTORC1 pathway may be a novel therapeutic strategy for age-related cardiovascular diseases.

#### 2.1.5 Metabolic and Hemodynamic Factors

In addition to the above triggers, major atherosclerotic risk factors such as hypertension and hyperglycemia also contribute to endothelial dysfunction. Hyperglycemia exerts its effects primarily through advanced glycation end-products (AGEs). The binding of AGEs to their receptor (RAGE) on human coronary artery ECs (HCAECs) activates the p38 and ERK1/2 signaling pathways, reduces eNOS expression and induces oxidative stress, ultimately leading to endothelial dysfunction [34]. Similarly, in mouse models, hyperglycemia has been shown to promote endothelial dysfunction by inducing the expression of functional adhesion molecules in the endothelium. Treatment with empagliflozin lowers blood glucose levels and reduces the expression of P-selectin, E-selectin, and VCAM-1 [35]. As a hemodynamic factor, hypertension shows a positive correlation between its severity and the extent of endothelial dysfunction. Under hypertensive conditions, sustained mechanical stress and oxidative stress lead to increased production of ROS, which exacerbates endothelial injury. Hypertension also activates the angiotensin II (Ang II) signaling pathway, inducing NF-κB and NLRP3 inflammasome activation, thereby promoting the release of pro-inflammatory mediators and disrupting the endothelial barrier [36].

### 2.2 Interaction Between Vascular Endothelium and Immune Cells

The interaction between ECs and immune cells comprises the core network that regulates vascular inflammation. A key regulator within this network is the transcription factor Gata6, which is highly expressed in healthy ECs. This was demonstrated in mouse models, where EC-specific deletion of *Gata6* resulted in significantly reduced monocyte infiltration and smaller atherosclerotic lesions. Mechanistically, Gata6 directly controls the target gene *Cytidine monophosphate kinase 2* (*Cmpk2*) that mediates immune cell recruitment. *Gata6* deletion lowers *Cmpk2* expression, which in turn reduces monocyte adhesion and inflammatory foam cell formation via the Cmpk2-Nlrp3 pathway. Gata6 also directly regulates another target, the chemokine C-C motif chemokine ligand 5 (CCL5), which is similarly involved in monocyte adhesion and migration [37]. These findings suggest that targeting Cmpk2 or CCL5 could be a novel therapeutic avenue for atherosclerosis, though further studies are needed to validate the clinical efficacy and safety of this approach.

Another protein involved in mediating the immune response in atherosclerosis is epithelial-stromal interaction 1 (EPSTI1). The expression of *EPSTI1* is significantly upregulated in human atherosclerotic plaques compared to healthy arteries. *In vitro* experiments have further clarified its role by demonstrating that overexpression of *EPSTI1* in HUVECs enhances THP-1 (an immortalized human monocytic cell line derived from an acute monocytic leukemia patient) monocyte adhesion through upregulation of the adhesion molecules VCAM-1 and ICAM-1 [38]. These findings suggest that EPSTI1 contributes to atherosclerosis by promoting monocyte recruitment to the endothelium, and therefore its targeting may represent a novel therapeutic strategy.

As a counterbalance to pro-inflammatory molecules, the endothelium also expresses protective proteins like endothelial-specific thrombomodulin (TM) that suppress excessive immune activation. The TM-thrombin complex catalyzes the conversion of protein C into its activated form (APC), which inhibits IKKβ phosphorylation via protease-activated receptor 1 (PAR1)/inhibitory G protein (Gi) coupling, blocking NF-κB nuclear translocation and the expression of downstream pro-inflammatory factors [39]. On the other hand, TM can also directly capture HMGB1 released by monocytes, preventing its interaction with TLR4/RAGE and thereby inhibiting NLRP3 inflammasome activation and IL-1β release to modulate the inflammatory phenotype of immune cells [37]. This anti-inflammatory mechanism is markedly impaired in *TM*-deficient models, manifesting as elevated basal NF-κB activity in ECs and as abnormal immune cell infiltration, suggesting it has a protective role in atherosclerosis and sepsis.

GlycoRNA is primarily found on the cell surface and plays a critical role in neutrophil recruitment *in vivo*. Recent studies have identified the presence of cell surface RNA (glycoRNA) on neutrophils, where it plays a critical role in inflammatory responses. The elimination of glycoRNA markedly diminishes the recruitment of neutrophils to inflammatory sites, as well as reducing their adhesion to and transmigration across ECs [40]. Another study showed that glycoRNA-coated neutrophil membrane-coated siMT1-loaded nanoparticles (GlycoRNA-NP-siMT1) can specifically deliver siMT1 to abdominal aortic aneurysm (AAA) lesions. GlycoRNA-NP-siMT1 mitigates pathological remodeling of the abdominal aorta by reducing neutrophil infiltration and inhibiting neutrophil extracellular trap (NET) formation [41], thus offering new possibilities for glycoRNA-targeted therapy. However, research on glycoRNA is still in its early stages, and its role in atherosclerosis has not been fully validated. Additional large-scale studies are needed to confirm its mechanism of action.

The preceding sections have detailed how diverse triggers, including abnormal hemodynamics, infection, and aging, contribute to endothelial dysfunction. These factors do not act in isolation but instead form a synergistic network, often converging on a few core pathological hubs such as the NF-κB pathway, the NLRP3 inflammasome, and the generation of ROS. A central feature of this network is a vicious cycle involving NO and ROS. The various upstream triggers all promote ROS production. Excess ROS not only causes direct cellular damage but also uncouples eNOS, creating a state of “NO-ROS imbalance” in which there is less production of protective NO. The decreased bioavailability of NO impairs vasodilation and removes its natural inhibitory effect on the NF-κB pathway, further intensifying inflammation. This creates a mutually reinforcing pathological loop of “infection-aging-shear stress-NO imbalance” that collectively drives sustained endothelial activation, increased permeability and leukocyte recruitment, ultimately initiating and accelerating the progression of atherosclerosis. The integrated model described above highlights the deep interconnectedness of these early disease mechanisms and suggests that multi-target therapeutic strategies may be particularly effective.

## 3. Lipid Accumulation and Inflammation

**Fig. 2.**
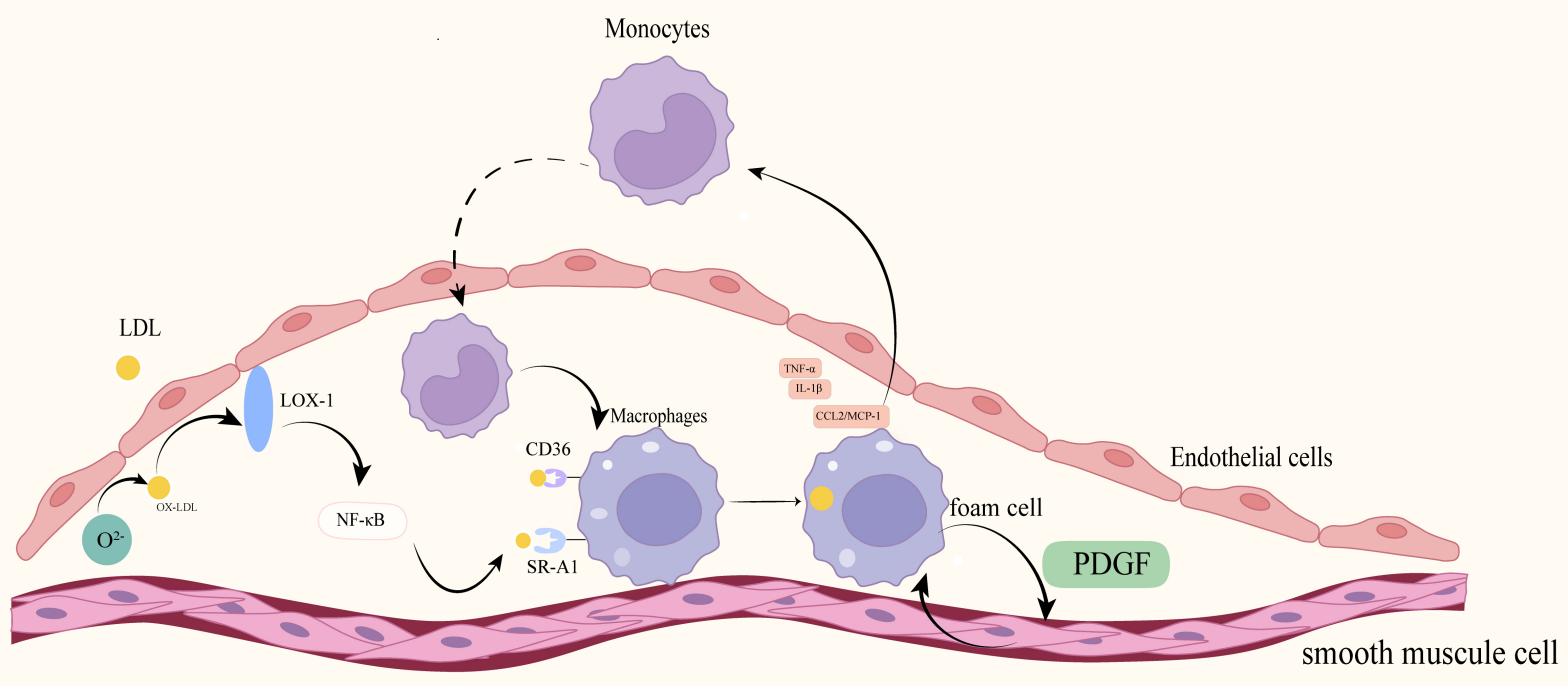
**Lipid accumulation and inflammation**. LDL particles are retained beneath the endothelium and undergo oxidative modification under conditions of oxidative stress and myeloperoxidase (MPO) catalysis, leading to the formation of oxidized low-density lipoprotein (ox-LDL). Ox-LDL binds to the lectin-like oxidized low-density lipoprotein receptor-1 (LOX-1) on endothelial cells (ECs), activating the NF-κB signaling pathway and subsequently inducing the expression of adhesion molecules and promoting the adhesion and transmigration of monocytes into the intima. Infiltrated monocytes differentiate into macrophages, which together with vascular smooth muscle cells uptake ox-LDL via scavenger receptors such as cluster of differentiation 36 (CD36) and scavenger receptor class A member 1 (SR-A1). This leads to the formation of foam cells and expansion of the lipid necrotic core. Foam cells and activated macrophages secrete pro-inflammatory cytokines (e.g., IL-1β, TNF-α) and chemokines (e.g., CCL2/MCP-1), further amplifying endothelial inflammation, monocyte recruitment, and lipid deposition. This establishes a vicious positive feedback loop between lipid accumulation and inflammation that drives plaque progression and instability. Fig. 2 was generated utilizing Adobe Illustrator 2025. PDGF, platelet-derived growth factor.

A defining feature of atherosclerosis is lipid accumulation, which begins when apolipoprotein B (apoB)-containing lipoproteins are retained within the arterial wall. Small lipoprotein particles, such as LDL and VLDL remnants, cross the endothelial barrier and enter the intima. Here, they are trapped by the extracellular matrix, leading to a high concentration of lipids within the vessel wall [42]. Lipid accumulation triggers a local inflammatory response, creating a self-perpetuating vicious cycle that drives the progression of atherosclerosis, as illustrated in Fig. 2 [43].

### 3.1 Vascular Smooth Muscle Cells (VSMCs) Phenotypic Switching and Dysregulation

The process of lipid accumulation and inflammation profoundly affects VSMCs, the principal component of the artery’s middle layer (tunica media). While they normally confer structural stability, VSMCs can also undergo a detrimental phenotypic switch in atherosclerosis, transforming them into a pro-inflammatory state and participating in lipid uptake [42]. The significance of this is highlighted by lineage-tracing studies, which show that up to 50% of foam cells in atherosclerotic plaques originate from transdifferentiated VSMCs [44]. This transformation is actively driven by lipids. One key mechanism, identified by *in vitro* studies using rat VSMCs and confirmed *in vivo* with mice, is the mtROS/c-Fos/lectin-like oxidized low-density lipoprotein receptor-1 (LOX-1) signaling axis. Ox-LDL stimulates mitochondrial ROS (mtROS), which activates the transcription factor c-Fos to upregulate the scavenger receptor LOX-1. This further increases lipid uptake, accelerating the conversion of VSMCs into foam cells [45]. Other pathways, such as cholesterol-induced reprogramming of the miR-143/145-myocardin axis, also contribute to this macrophage-like transformation [46].

The behavior of VSMCs within the plaque is governed by a complex network of counteracting signals. While some pathways promote their harmful transformation, others offer crucial protection. For instance, the serine-threonine kinase Akt1 acts as a key survival signal by suppressing the pro-apoptotic factor forkhead box protein O3a (FoxO3a), thereby protecting VSMCs from cell death and mitigating adverse arterial remodeling [47]. Another key protective factor is the nuclear deacetylase SIRT6, whose expression in VSMCs is markedly reduced in both human and murine atherosclerotic plaques. Mechanistically, SIRT6 prevents VSMC senescence by maintaining telomere integrity, a function that is dependent on its deacetylase activity. The importance of this mechanism was confirmed in a preclinical study on apolipoprotein E (ApoE)^-⁣/-^ mice, wherein VSMC-specific overexpression of functional SIRT6 reduced atherosclerosis, but overexpression of a deacetylase-deficient version worsened features of plaque instability [48].

Similarly, the extracellular matrix protein CCN2 secreted by VSMCs has been identified as another protective regulator. In mouse models, SMC-specific deletion of CCN2 resulted in larger atherosclerotic lesions that showed elevated endoplasmic reticulum stress (ERS) and increased lipid uptake. Single-cell analyses suggest that CCN2 helps to maintain a healthy VSMC phenotype by suppressing an ERS-endocytosis axis that would otherwise promote a harmful macrophage-like transformation [49]. Conversely, defects in protective mechanisms like autophagy can amplify VSMC death and accelerate disease progression.

These findings provide supportive evidence for the role played by VSMCs in atherosclerosis. Understanding the balance between the various detrimental pathways and the impaired protective pathways is crucial for developing therapies aimed at maintaining a healthy VSMC phenotype.

### 3.2 Regulation of Lipophagy

A key cellular process for managing the lipid accumulation that drives VSMC transformation is lipophagy, a specialized form of autophagy that degrades lipid droplets. This process involves enclosing lipid droplets in autophagosomes, which then fuse with lysosomes where enzymes break down the lipids. The resulting free cholesterol can then be used by the cell, or removed via the ATP-binding cassette transporter G1 (ABCG1) transporter [50, 51]. Lysosomal pH imbalance or diminished cathepsin activity can obstruct autophagic flux, leading to abnormal lipid droplet accumulation. Properly functioning lipophagy is critical for lipid homeostasis, and its impairment is a shared pathogenic mechanism in metabolic diseases like atherosclerosis [52]. Recent preclinical research has identified a specific regulator of this process in VSMCs, the P2RY12 receptor, which acts as a significant suppressor of autophagy. Mechanistic studies have shown that P2RY12 inhibits key steps in the autophagic machinery, including maturation of MAP1LC3/LC3 (Microtubule-Associated Protein 1 Light Chain 3), a key protein marker of autophagy. The importance of this mechanism was confirmed in ApoE^-⁣/-^ mouse models, where pharmacological blocking of the P2RY12 receptor enhanced VSMC autophagy and consequently reduced the progression of atherosclerosis [53].

Acid-sensing ion channel 1 (ASIC1) influences lipophagy by impeding cholesterol efflux in macrophages [54]. *In vitro* experiments have shown that ASIC1 activation promotes phosphorylation of the signaling protein receptor-interacting protein 1 (RIP1, also known as RIPK1**)** and the master autophagy regulator, transcription factor EB (TFEB). In cellular models, phosphorylation of TFEB hindered its nuclear translocation and suppressed the expression of essential lysosomal genes, ultimately disrupting the lipophagy process and causing lipid accumulation [55]. Based on these findings, the targeting of regulatory pathways such as the P2RY12 axis in VSMCs and the ASIC1-TFEB axis in macrophages has been proposed as a novel therapeutic strategy. However, significant further research is required to validate these specific mechanisms in the human context and to assess the long-term safety and efficacy of such targeted interventions.

### 3.3 Reverse Cholesterol Transport and Atherosclerosis

Building on the concept of cellular lipid clearance, reverse cholesterol transport (RCT) is a critical systemic process in which macrophages remove cholesterol from the arterial wall using a pathway that is heavily dependent on the transporter ABCA1 [56]. The regulation of RCT is complex and involves various factors, including non-coding RNAs. In mouse models, the long non-coding RNA (lncRNA) AI662270 was found to inhibit cholesterol efflux by directly attenuating the expression and activity of ABCA1 [57]. Conversely, lncRNA PCA3 was found to be downregulated in cellular studies of ox-LDL-induced foam cells, whereas miR-140-5p was highly expressed. A possible mechanism is that lncRNA PCA3 increases expression of the transcription factor regulatory factor X7 (RFX7, a transcription factor) and ABCA1 by competitively binding miR-140-5p, thus promoting cholesterol efflux [58].

Several other molecules have also been shown to regulate the RCT pathway. The gut microbiota metabolite indole-3-propionic acid (IPA) promotes RCT in preclinical models, reportedly through the miR-142-5p/ABCA1 pathway [59]. Similarly, the adipokine Asprosin was shown to increase cholesterol efflux in cellular and mouse models by activating the p38/Elk-1 signaling cascade to boost the transcription of ABCA1 and ABCG1 [60].

Conversely, other factors can impair RCT. One such molecule is Tumor necrosis factor-alpha-induced protein 1 (TNFAIP1), initially identified as being induced by TNF-α and LPS in umbilical vein ECs [61]. TNFAIP1 was found to promote inflammatory responses and oxidative stress in atherosclerosis [62]. Xu *et al*. [63] reported that TNFAIP1 epigenetically silences the expression of lncRNA LEENE, which in turn prevents degradation of the transcription factor forkhead box protein O1 (FoxO1). The resulting accumulation of FoxO1 then suppresses transcription of ABCA1, leading to reduced cholesterol efflux and increased lipid accumulation [63]. While not a direct study of atherosclerosis, a preclinical model of acute ischemia-reperfusion injury found that knockdown of TNFAIP1 ameliorates myocardial damage and inflammation [64]. However, it is important to distinguish between the pathophysiology of acute ischemia-reperfusion injury and that of chronic atherosclerotic plaque development. Therefore, the specific role and therapeutic potential of targeting TNFAIP1 in atherosclerosis requires direct investigation and validation.

Adding to the complexity of molecular regulation in atherosclerosis, the lipid-sensing receptor triggering receptor expressed on myeloid cells 2 (TREM2) found on macrophages appears to have multifaceted and sometimes contradictory roles [65]. On the one hand, some research has indicated a detrimental function. For example, Guo *et al*. [66] reported that overexpression of TREM2 in macrophages upregulated the scavenger receptor cluster of differentiation 36 (CD36), which then increased lipid uptake and the formation of foam cells. On the other hand, TREM2-deficient macrophages have lower survival and impaired phagocytosis under lipid-loading conditions, thereby exacerbating necrotic core formation. Conversely, TREM2 activation protects against atherosclerosis by limiting necrotic core formation [66]. This apparent discrepancy demonstrates the highly context-dependent function of TREM2, which likely varies with the disease stage or the plaque micro-environment. Therefore, future therapeutic strategies targeting TREM2 must be highly nuanced and involve stage- or cell-specific modulation rather than uniform activation or inhibition.

Sirtuin 6 (Sirt6) is a histone deacetylase that enhances plaque stability by promoting macrophage autophagy and lipophagy [67]. It achieves this partly by inhibiting Wnt1/β-catenin signaling, and potentially also by upregulating the liver X receptor α (LXRα)/ABCA1 cholesterol efflux pathway [68]. Underscoring its importance, macrophage-specific knockdown of Sirt6 was shown to increase scavenger receptor expression and lipid uptake both *in vitro* and *in vivo*, thus promoting a pro-atherogenic phenotype [69]. Sirt6 activity in VSMCs is increased by the kinase liver kinase B1 (LKB1, also known as Serine/threonine kinase 11). This LKB1-mediated activation of Sirt6 inhibits the scavenger receptor LOX-1, thereby reducing lipid uptake and the formation of VSMC-derived foam cells [70]. These findings provide a strong rationale for exploring Sirt6 activation as a potential therapeutic strategy, although further research is required before translation into the clinic.

### 3.4 CD36 Palmitoylation-Mediated Lipid Accumulation and Inflammation

CD36 is a scavenger receptor regulated by palmitoylation. It plays a key role in atherosclerosis by acting as a receptor for pro-atherosclerotic, oxidized high-density lipoprotein (ox-HDL) [71, 72, 73]. HDL is a cholesterol carrier that mediates RCT [74]. Preclinical studies, primarily *in vitro*, have shown that ox-HDL can catalyze the palmitoylation of CD36, causing it to cluster in lipid raft microdomains. This single modification is thought to initiate a vicious cycle in which raft-localized palmitoylated CD36 not only increases the uptake of ox-HDL, but also simultaneously triggers pro-inflammatory signaling via the c-Jun N-terminal kinase (JNK) cascade and impairs lipid droplet clearance by inhibiting autophagy [75]. Furthermore, co-immunoprecipitation experiments suggest that palmitoylated CD36 forms a complex with the innate immune receptor TLR4. Inhibition of this CD36-TLR4 interaction was observed to reduce lipid accumulation [76].

In essence, these findings indicate that CD36 palmitoylation is a critical molecular switch that can transform the CD36 receptor into a potent driver of both lipid accumulation and inflammation.

### 3.5 Lipid Homeostasis and Atherosclerosis

The intracellular breakdown of lipids is carried out by key enzymes such as adipose triglyceride lipase (ATGL). This enzyme binds to the surface of lipid droplets (LDs) and catalyzes the breakdown of triglyceride (TG) to release free fatty acids (FAs) [77]. In atherosclerosis models, endothelial deficiency of ATGL has been linked to vascular lipid accumulation and dysfunction. The proposed mechanism involves the induction of ERS by TG accumulation, which in turn promotes inflammation via the NF-κB pathway and impairs NO production by inhibiting eNOS [78]. Recent cellular studies have uncovered an upstream pathway that regulates ATGL. The transcription factor X-box binding protein 1, spliced form (XBP1s) increases the expression of ER degradation-enhancing α-mannosidase-like protein 2 (EDEM2), which then acts with its partner secretory 23 homolog A (SEC23A) to promote the localization of ATGL to lipid droplets where it is protected from degradation [79]. While the XBP1s-EDEM2-ATGL axis is an important regulator of cardiac lipid homeostasis, its specific role in the endothelial dysfunction of atherosclerosis requires further investigation.

### 3.6 Kindlin3 and Atherosclerosis

The atherosclerotic environment also disrupts the function of the adhesome, a protein complex that regulates cell adhesion, with Kindlin3 (K3) being a vital component for macrophage function. *In vitro* studies with macrophage cultures show that ox-LDL reduces K3 levels. This weakens the K3-integrin β1 interaction, which in turn upregulates the scavenger receptor LOX-1 and further enhances ox-LDL uptake [80]. However, K3 has been shown to have a beneficial role in a different disease context. In a mouse model of myocardial infarction (MI), overexpression of K3 promoted new blood vessel formation and reduced cardiac fibrosis and cardiomyocyte apoptosis, an effect mediated through the Notch signaling pathway [81]. These findings suggest that K3 is a key regulator of macrophage function and consequently merits further investigation as a potential therapeutic target. The concept of restoring K3 expression to slow disease progression is compelling, but its feasibility, long-term effects, and potential off-target consequences require extensive preclinical investigation.

### 3.7 Integration of Lipid Signaling and Inflammatory Pathways

Beyond lipid accumulation, specific molecular axes that tightly couple lipid metabolism with inflammatory signaling are now understood to be critical drivers of atherosclerosis. These regulatory hubs operate at multiple cellular levels.

Proprotein convertase subtilisin/kexin type 9 (PCSK9) negatively modulates the low-density lipoprotein receptor (LDLR) via lysosomal degradation, while also directly promoting macrophage inflammatory responses [82]. Moreover, PCSK9 was found to increase TLR4/NF-κB signaling in cellular models [83]. Cholesterol accumulation resulting from LDLR deficiency was shown to activate the NLRP3 inflammasome in mice, promoting IL-1β/IL-18 secretion and thereby accelerating plaque progression [84].

Another key node that acts within the cytoplasm is the ADP-ribosylation factor-like protein 11 (ARL11)/Janus kinase 2 (JAK2)/STAT1 axis. ARL11 is an ADP-ribosylation factor-like GTPase that activates JAK2 to promote STAT1 phosphorylation. It is highly expressed in atherosclerotic plaques [85], and its silencing was observed to reduce both lipid deposition and plaque area in atherosclerosis models. The ARL11 signaling cascade has also been shown to drive pro-inflammatory M1 macrophage polarization [86].

The protein perilipin 1 (PLIN1) is a crucial protective gatekeeper at the lipid droplet surface. Dysfunction of this lipid droplet-coating protein can lead to ectopic lipid deposition and metabolic inflammation. Evidence from clinical genomics studies has shown that deficiency of PLIN1 promotes inflammatory cytokine secretion, while its overexpression in cellular models inhibits ox-LDL uptake and increases cholesterol efflux [87]. Underscoring its clinical relevance, human genome-wide association studies (GWAS) have linked loss-of-function PLIN1 mutations to increased coronary artery calcification scores [88, 89].

In summary, we have described how lipid accumulation in atherosclerosis is far from a passive process. It triggers a cascade of cellular dysfunctions, most notably the pro-inflammatory phenotypic switching of VSMCs. The maintenance of cellular lipid homeostasis depends on a delicate balance between lipid uptake, lipophagy, and cholesterol efflux through RCT. These processes are tightly controlled by a complex network of molecular regulators that include non-coding RNAs, signaling kinases, cell surface receptors, and lipid droplet-associated proteins. Disruption of these regulatory axes provides a direct mechanistic link between disordered lipid metabolism and the amplification of inflammatory responses.

## 4. Inflammatory Biomarkers

The inflammatory response is now recognized as the central driving force throughout the entire progression of atherosclerosis [90]. Infiltrating plasma lipoproteins undergo modifications that activate resident inflammatory cells, which then release inflammatory signals that recruit more circulating leukocytes to the site, further amplifying the response and establishing a “lipid infiltration-inflammation activation-cell recruitment” positive-feedback loop [91]. This chronic inflammation involves intricate cross-talk between the innate and adaptive immune systems, ultimately driving plaque progression and leading to clinical cardiovascular events.

### 4.1 Core Regulation of Pro-Inflammatory Factors

The inflammatory response in atherosclerosis is orchestrated by several core signaling hubs. This review will focus on key nodes in the inflammatory signaling pathways that function as master regulators of the downstream inflammatory cascade, including the NLRP3 inflammasome and major cytokines such as IL-1β and IL-6.

NLRP3-IL-1β/IL-18 Axis: Research has shown the NLRP3 inflammasome correlates with disease severity in acute coronary syndrome (ACS) patients, with elevated plasma NLRP3 levels being associated with poor short-term ACS prognosis [92]. In atherosclerotic mouse models with clonal hematopoiesis, inhibition of NLRP3 or IL-1β leads to increased fibrous cap formation and enhanced plaque stability [93].

IL-6 Signaling Network: In atherosclerosis, the IL-6/signal transducer and activator of transcription 3 (STAT3) axis promotes the expression of endothelial adhesion molecules, increases monocyte adhesion and foam cell formation, and induces the proliferation of smooth muscle cells and MMP expression, thereby destabilizing plaques. Proteomic analyses have identified CXCL10 as a downstream mediator of IL-6 that recruits CD8^+^T cells and pro-inflammatory CD68^+^CD163^–^ macrophages to the plaque shoulder, leading to increased plaque vulnerability [94].

**Fig. 3.**
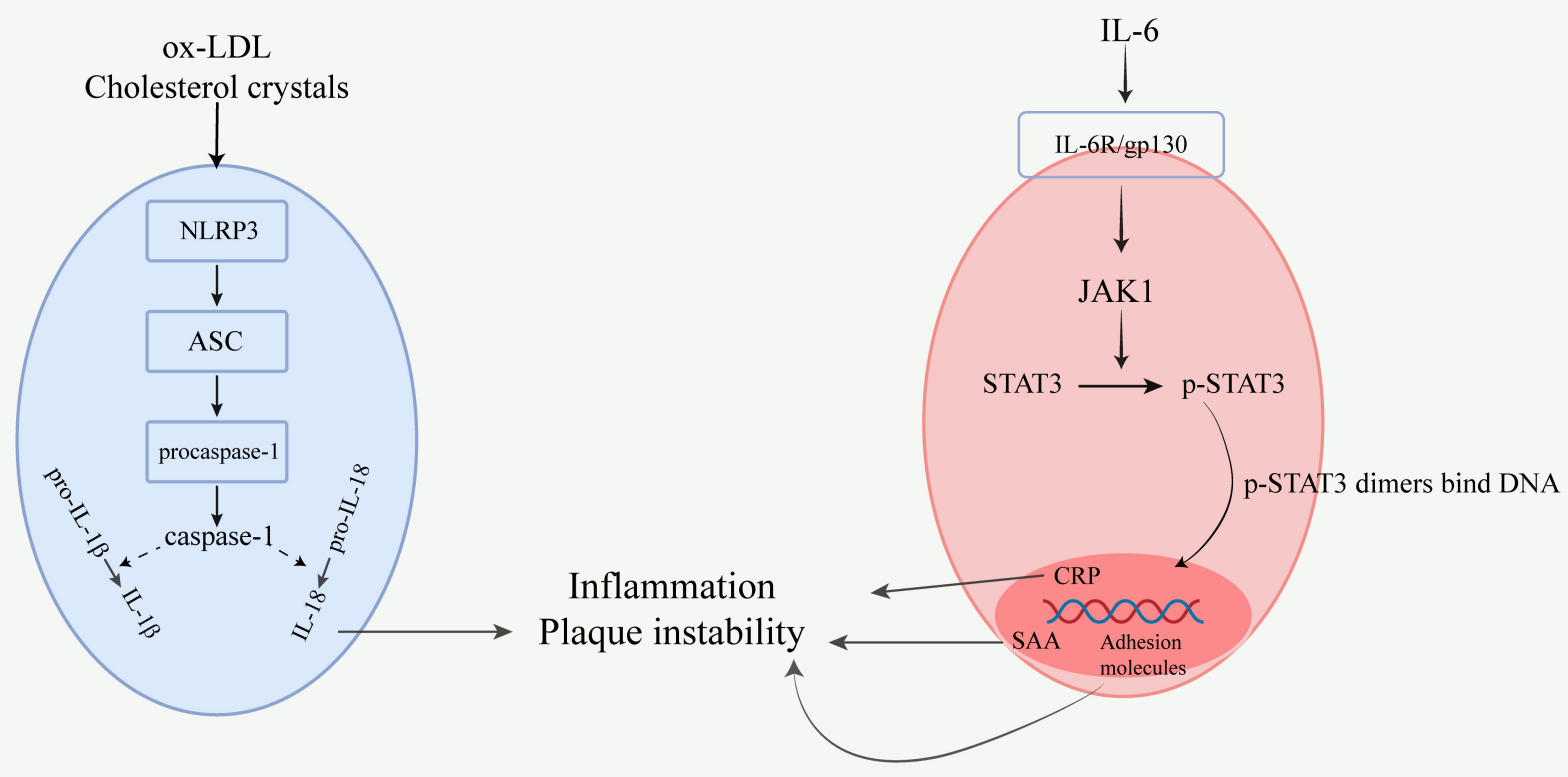
**Mechanisms involving the NLRP3 inflammasome and IL-6/STAT3 pathway in atherosclerosis**. In atherosclerosis, the NLRP3 inflammasome is activated by ox-LDL and cholesterol crystals, leading to the assembly of NLRP3 with ASC (apoptosis-associated speck-like protein) and procaspase-1 into an inflammasome complex. This process activates caspase-1, which cleaves pro-IL-1β and pro-IL-18 to release their mature forms, IL-1β and IL-18, respectively. These cytokines exacerbate inflammation through the NF-κB and mitogen-activated protein kinase (MAPK) signaling pathways, upregulate the expression of matrix metalloproteinases (MMPs), and degrade the fibrous cap of atherosclerotic plaques, thereby increasing the risk of plaque rupture. Concurrently, ECs, macrophages, and T cells within the lesion secrete IL-6, which binds to the IL-6R/gp130 receptor complex, activating Janus kinase 1 (JAK1) and phosphorylating signal transducer and activator of transcription 3 (STAT3). The p-STAT3 dimers translocate to the nucleus, where they induce the expression of C-reactive protein (CRP), serum amyloid A (SAA), and adhesion molecules (e.g., ICAM-1 and VCAM-1), promoting leukocyte infiltration and amplifying the inflammatory response. Fig. 3 was generated utilizing Adobe Illustrator 2025.

Mechanisms involving the NLRP3 inflammasome and IL-6/STAT3 pathway in atherosclerosis (Fig. 3).

IL-1β/IL1RAP Regulatory Circuit: IL-1 receptor accessory protein (IL1RAP) is a co-receptor for IL-1 family signaling. The IL-1β-induced signaling pathway is initiated through a receptor complex consisting of the ligands IL-1β, IL-1 receptor type 1 (IL1R1), and IL1RAP that regulates inflammatory and immune responses. IL1RAP is highly expressed in myeloid cells within human plaques and plays a pivotal role in atherosclerosis. In atherosclerotic mouse models, anti-IL1RAP therapy markedly reduces the macrophage content of plaques, with trends toward decreased neutrophil and T cell infiltration. Specifically, IL1RAP blockade reduces the arterial expression of leukocyte-recruiting chemokines (e.g., CXCL1, CXCL2, CXCL5) and adhesion molecules, and ameliorates endothelial function by inhibiting NF-κB/AP-1-mediated NET formation [95]. Angiopoietin-like protein 3 (ANGPTL3) can interact with IL1R1 and IL1RAP to disrupt the signaling complex assembly, thereby inhibiting NF-κB activation [96].

### 4.2 Spatial Regulation of Chemokines

This section will focus on the spatial dynamics of inflammation by exploring how chemokines and their receptors orchestrate the precise recruitment and positioning of immune cells within the local microenvironment of the atherosclerotic plaque.

CCL2-CCR2 Axis: Endothelial denudation and pro-thrombotic components of atherosclerotic plaques can trigger chemokine release [97]. Preclinical studies have shown that C-C chemokine receptor type 2 (CCR2) antagonists reduce plaque IL-1β levels and inhibit macrophage infiltration, highlighting CCR2 as a key target for anti-inflammatory intervention [98].

CCL5/RANTES: CCL5/RANTES is a chemokine that mediates the chemotaxis and activation of T cells, monocytes, mast cells, and dendritic cells. It interacts with chemokine receptors (CCR1, CCR3, CCR4, and CCR5) and plays a key role in the inflammatory process [99]. In an accelerated atherosclerosis model, CCR5 antagonism promoted the formation of stable plaques with fewer macrophages [100]. Treatment with HT-C6, a synthetic derivative of the natural olive antioxidant hydroxytyrosol, has been shown in cellular models to inhibit NF-κB pathway activation and CCL5 expression in the endothelium, thereby suppressing endothelial inflammation [101]. However, low levels of CCL5 following ST-segment elevation MI were found to be associated with an increased risk of MACE [102]. Future research should further explore the roles of CCL5 in different stages of atherosclerosis and its interactions with other molecules.

Pathological Effects of HCC-1 (CCL14): Hemofiltrate chemokine 1 (HCC-1), also known as CCL14. Clinical and bioinformatic analyses have shown that HCC-1 expression is elevated in the serum and atherosclerotic plaques of patients. Moreover, it is positively correlated with disease occurrence and the switch from stable to unstable plaques. In ApoE^-⁣/-^ mice, the overexpression of HCC-1 increased inflammatory factors, macrophage accumulation, and pyroptosis within plaques, thereby decreasing their stability. Complementary cellular studies demonstrated that HCC-1 directly promotes pro-atherogenic processes such as monocyte adhesion and pro-inflammatory M1 macrophage polarization. These effects are reportedly mediated through the NF-κB/NLRP3/Caspase-1 signaling pathway [103]. Given its strong association with disease progression, HCC-1 is a promising biomarker for diagnosis and risk stratification, and its targeting may offer a novel therapeutic approach for atherosclerosis.

The growing understanding of the central role played by inflammation in atherosclerosis has spurred the development of targeted, anti-inflammatory therapies. The CANTOS trial, for instance, provided pivotal proof-of-concept by demonstrating that targeting of the IL-1β pathway with canakinumab could significantly reduce cardiovascular events, independent of lipid-lowering effects. This success solidified the “inflammation hypothesis” of atherosclerosis and opened the way for targeting other cytokine pathways. Subsequent research has explored agents targeting the IL-6 axis, such as tocilizumab, which has shown promise by improving vascular function in acute settings. Besides the major cytokines, the complex network of chemokines, which orchestrate leukocyte trafficking, presents another attractive set of targets. Preclinical studies targeting axes such as CCL2-CCR2 and CCL5-CCR5 have shown success in reducing macrophage infiltration and plaque size in animal models. However, translating these findings to human therapies remains challenging due to the potential effects on systemic immunity.

Table 1 (Ref. [97, 104, 105, 106, 107, 108, 109, 110, 111, 112, 113, 114, 115, 116, 117, 118, 119]) provides a comprehensive summary of these and other key inflammatory mediators, detailing their specific mechanisms, therapeutic agents that target them, and the current status of clinical or preclinical evidence for intervention.

**Table 1.** **Key inflammatory mediators in atherosclerosis: mechanisms and therapeutic progress**.

Mediator	Core mechanism	Clinical/Preclinical intervention status
NLRP3	Ox-LDL and cholesterol crystals activate the NLRP3 inflammasome, leading to caspase-1 activation and the release of IL-1β and IL-18, which drive inflammatory responses in ECs and macrophages [104].	MCC950 (a small-molecule NLRP3 inhibitor) significantly reduces plaque burden in atherosclerotic mouse models [107].
IL-1β	Activation of the NLRP3 inflammasome triggers IL-1β release, activating STAT3/NF-κB signaling and promoting expression of adhesion molecules and formation of foam cells.	CANTOS trial: Canakinumab (anti-IL-1β) significantly reduces the risk of cardiovascular events [108].
IL-6	IL-6R/gp130 activates the JAK1/STAT3 pathway, inducing acute-phase proteins (CRP, SAA) and adhesion molecules, thereby exacerbating endothelial inflammation and macrophage polarization [105].	Tocilizumab (anti-IL-6R) has shown improvement of vascular function in a small-scale trial of acute myocardial infarction [109].
TNF-α	TNF-α activates NF-κB and JNK via tumor necrosis factor receptor 1 (TNFR1), promoting endothelial apoptosis, adhesion molecule expression, and macrophage inflammatory responses.	Anti-TNF-α biologics (e.g., infliximab) improve vascular function and lower cardiovascular risk in rheumatoid arthritis patients [110], although large-scale atherosclerotic cardiovascular disease (ASCVD) trials are lacking.
IL-17A	Th17 cells secrete IL-17A, inducing ECs and macrophages to produce IL-6, TNF-α, and CCL2, thereby promoting inflammatory cell infiltration and plaque instability.	Secukinumab (anti-IL-17A) shows neutral effects on vascular inflammation and cardiometabolic biomarkers in psoriasis patients [111]. ASCVD studies are ongoing.
IL-23	IL-23 drives Th17 cells to secrete IL-17A and promotes inflammatory activation of macrophages and expression of matrix-degrading enzyme MMP-9 via the STAT3/retinoic acid-related orphan receptor gamma t (RORγt) pathway, thus aggravating plaque instability.	Anti-IL-23 monoclonal antibodies (e.g., tildrakizumab) have demonstrated safety in autoimmune disease patients [112]. Investigation of their potential for cardiovascular protection is being evaluated.
CCL2-CCR2	CCL2 binds to CCR2 to mobilize Ly6C^high^ monocytes from the bone marrow and guide their infiltration into plaques, increasing pro-inflammatory gene expression in macrophages [97].	Inhibiting the CCL2/CCR2 axis may stabilize atherosclerotic plaques and reduce complications such as acute coronary syndromes [113].
CCL5-CCR5	CCL5 recruits CCR5^+^ monocytes and T cells into plaques, promotes M1 polarization of macrophages, and induces MMP-2/9 expression, leading to matrix degradation.	Maraviroc (CCR5 antagonist) reduces plaque size and inflammation in the ritonavir-induced atherosclerosis model [114]. It also inhibits NADPH oxidase 1 (Nox1) expression to reduce vascular inflammation [115].
CXCL8 (IL-8)	IL-8 is secreted by ECs and macrophages and promotes the chemotaxis of neutrophils and monocytes via C-X-C chemokine receptor (CXCR) 1/2, enhancing ROS production and expression of adhesion molecules.	Reparixin (CXCL8 receptor antagonist) reduces endothelial damage in an *in vitro* model of ischemia-reperfusion [116].
CXCL10-CXCR3	CXCL10 recruits CXCR3^+^ Th1 cells and natural killer cells (NK) to the lesion site, where they secrete IFN-γ, exacerbating macrophage activation and matrix degradation.	CXCR3 antagonists block the direct migration of CXCR3^+^ effector cells into plaques, modulate inflammatory responses, and reduce arterial inflammation [117].
CXCL12-CXCR4	CXCL12/CXCR4 activates glycogen synthase kinase 3 beta (GSK3β)/β-catenin^T120^/transcription factor 21 (TCF21), downregulating ABCA1, inhibiting cholesterol efflux, and exacerbating foam cell formation [106].	CXCR4 inhibitor AMD3100 blocks abdominal aortic aneurysm expansion and rupture in mice, and reduces inflammation and immune cell accumulation [118].
CX3CL1-CX3CR1	CX3CL1 (fractalkine) is expressed by activated ECs and macrophages, mediating adhesion and chemotaxis to the lesion site via CX3CR1 on CD14^+^CD16^–^ monocytes. Overactivation of this axis is associated with increased susceptibility to coronary atherosclerosis.	CX3CR1 antagonist KAND567 (an antagonist of the human CX3CR1 receptor) blocks CX3CL1 signaling, reducing post-myocardial infarction, immune cell recruitment and inflammation, and decreasing infarct size [119].
CCL20-CCR6	CCL20 is highly expressed in atherosclerotic plaques, binding to CCR6 to promote the migration of Th17 cells and CCR6^+^ monocytes to the lesion site, and facilitating matrix degradation and the spread of inflammation through MMP-2/9.	CCR6 antagonists remain in the preclinical research stage.

ABCA1, ATP-binding cassette transporter A1.

Collectively, the various cytokines and chemokines discussed above form a dynamic and complex inflammatory interactome that plays a central role in the microenvironment of the atherosclerotic plaque. A clear regulatory hierarchy exists within this network. For instance, upstream signaling hubs centered on the NLRP3 inflammasome can, upon activation, drive maturation and release of the key downstream effectors IL-1β and IL-18, which in turn trigger a cascade that amplifies the production of other cytokines such as IL-6. Concurrently, the chemokine network (e.g., the CCL2-CCR2 axis) spatially and precisely regulates the recruitment of immune cells, translating systemic inflammatory signals into local cellular infiltration and tissue remodeling. Therefore, the inflammatory microenvironment of atherosclerosis is not driven by isolated molecules, but is instead the result of a multi-layered, interconnected signaling network that maintains and amplifies the pathological state.

## 5. MicroRNAs: Key Regulatory Nodes Connecting Core Pathological Mechanisms

Operating at the post-transcriptional level, microRNAs (miRNAs) function as systemic integrators in the complex regulatory network of atherosclerosis, acting as molecular bridges that connect core pathological processes. For instance, the lipid-centric miR-33, co-transcribed with its host gene *SREBP*, links cholesterol synthesis with the inhibition of cholesterol efflux by targeting ABCA1, while also promoting pro-inflammatory macrophage polarization [120]. The classic “inflamma-miR”, miR-155, creates a vicious cycle by translating inflammatory signals into endothelial injury via eNOS suppression, and further inflammation via NF-κB and NLRP3 activation [121]. In contrast, miR-146a represents a negative feedback counterbalancing mechanism within the network. It is induced by NF-κB, which in turn inhibits the pathway by targeting interleukin-1 receptor-associated kinase 1 (IRAK1) and tumor necrosis factor receptor-associated factor 6 (TRAF6), effectively “pumping the brakes” on inflammation [122]. Therefore, the progression of atherosclerosis can be viewed in part as an imbalance within this network of functionally diverse miRNAs, where pro-inflammatory signals overwhelm their anti-inflammatory counterparts.

## 6. Sex Differences in Atherosclerosis

The incidence and complications of atherosclerosis exhibit sex dimorphism. Due to the protective effects of estrogen against atherosclerosis, the onset of CAD in women is delayed by 10–15 years compared to men. After menopause, traditional risk factors such as hypertension, dyslipidemia, and diabetes have a greater impact on the development of CVD in women [123]. Sex differences are also observed in plaque size, composition, and rupture risk. Premenopausal women tend to develop stable, diffuse lesions, whereas men are more prone to acute plaque rupture. In postmenopausal women, the triggering mechanisms for ACS may be more closely associated with systemic endothelial dysfunction and a hypercoagulable state [124]. These variations extend to the genetic level. A Study using reproductive models has found that, compared with the *XY* genotype, the *XX* genotype upregulates key enzymes involved in both free radical scavenging during injury and processes common to celiac disease, thereby enhancing the bioavailability of dietary fats [125]. This, in turn, provides the material basis for elevated blood lipids and plaque formation. Such findings suggest that the biological basis of women may not be inherently “protected”, but they may instead have a higher genetic predisposition to dyslipidemia. This underscores the need to adopt a sex-specific perspective in future research and therapeutic strategies.

## 7. Conclusion and Future Outlook

Atherosclerosis is a chronic inflammatory disease driven by endothelial dysfunction, lipid metabolism disorders, and persistent inflammation. It remains the leading cause of cardiovascular disease incidence and mortality worldwide. Here, we summarize the complex interactions among the core mechanisms, highlighting how key molecular pathways collaboratively regulate plaque initiation, progression, and rupture. Furthermore, we explore emerging regulatory factors such as glycoRNA and HCC-1, which provide new insights into the modulation of inflammation and also demonstrate potential as novel diagnostic biomarkers.

Future research will extend beyond single molecules or pathways, and focus instead on revealing the full spectrum of the disease through advanced research tools. Multi-omics integration techniques that combine genomics, transcriptomics, proteomics, and metabolomics data offer an unprecedented multidimensional molecular profile of the disease, enabling in-depth phenotyping of patients. This approach not only uncovers the complete flow of molecular information from static genetic risks (genotype) to dynamic disease manifestations (phenotype), but also facilitates the discovery of novel biomarker combinations associated with plaque instability [126]. At the same time, artificial intelligence (AI) and machine learning (ML) have revolutionized our ability to analyze these vast and complex datasets. AI/ML algorithms excel in developing cardiovascular risk prediction models that outperform traditional scoring systems, such as QRISK3 and ASCVD/PCE [127, 128]. They have also achieved groundbreaking advances in cardiovascular imaging. In particular, deep learning-based radiomics can extract subtle texture features from standard anatomical images, such as coronary computed tomography angiography. Such features are imperceptible to the human eye and are related to inflammation in perivascular adipose tissue (PCAT), enabling non-invasive quantification of local vascular inflammation [129].

On the therapeutic front, future strategies are likely to become more targeted and intelligent. Nanomedicine offers innovative solutions to the delivery challenges of nucleic acid drugs, such as antisense oligonucleotides and siRNA. Nanocarriers constructed from materials like chitosan and gold nanoparticles can precisely deliver drugs to atherosclerotic plaques [130]. Furthermore, “smart” delivery systems, such as pH-low insertion peptides (pHLIP), can utilize the acidic microenvironment of plaques to trigger targeted drug release to specific cells such as macrophages within the lesion (e.g., the delivery of anti-miR-33) [131]. This strategy integrates diagnosis with treatment, thereby maximizing efficacy while minimizing systemic side effects, and heralding the era of “theranostics”.

In summary, our improved understanding and management of atherosclerosis is leading to a new era of multidimensional, systematic, and personalized approaches, thanks to the clarification of core pathological mechanisms, the application of cutting-edge research tools, and the development of precision treatment strategies. Collectively, these advances hold great promise for ameliorating the management of atherosclerosis, achieving true individualized treatment, and ultimately reducing its global health burden.
